# Renal Biomarkers and Albuminuria Predict Early Adverse Outcomes in Cardiorenal Syndrome Type 2

**DOI:** 10.3390/medsci14020163

**Published:** 2026-03-25

**Authors:** Minela Bećirović, Emir Bećirović, Emir Begagić, Kenana Ljuca, Amir Bećirović, Denis Mršić, Nadina Ljuca, Mugdim Bajrić, Farid Ljuca

**Affiliations:** 1 Internal Medicine Clinic, University Clinical Center Tuzla, 75000 Tuzla, Bosnia and Herzegovina; minela03@live.com (M.B.); amirbecirovictz@gmail.com (A.B.); drdenis.m@gmail.com (D.M.); 2 Department of Neurosurgery, Cantonal Hospital Zenica, 72000 Zenica, Bosnia and Herzegovina; begagicem@gmail.com; 3 Department of Gynecology and Obstetrics, University Clinical Center Ljubljana, 1000 Ljubljana, Slovenia; kenana.ljuca.medf@gmail.com; 4 School of Medicine, University of Tuzla, 75000 Tuzla, Bosnia and Herzegovina; ljucanadina@gmail.com; 5 Clinic for Invasive Cardiology, University Clinical Center Tuzla, 75000 Tuzla, Bosnia and Herzegovina; dr.mugdim.bajric@gmail.com; 6 Department of Physiology, School of Medicine, University of Tuzla, 75000 Tuzla, Bosnia and Herzegovina; farid.ljuca@gmail.com

**Keywords:** cardiorenal syndrome type 2, heart failure, cystatin C, albuminuria, renal biomarkers, prognosis

## Abstract

**Background/Objectives:** Cardiorenal syndrome type 2 (CRS-2) is characterized by progressive renal dysfunction caused by chronic heart failure (HF) and is associated with increased morbidity and mortality. However, the prognostic value of renal biomarkers in patients with CRS-2 hospitalized for decompensated HF remains unclear. **Methods:** This prospective observational cohort study included 200 consecutive patients hospitalized for decompensated HF in the Intensive Care Unit of the Clinic for Internal Medicine at the University Clinical Centre Tuzla between April and October 2025. CRS-2 was defined as chronic HF with chronic kidney disease persisting for ≥3 months before admission according to KDIGO criteria. Patients were followed for three months. The primary composite outcome was all-cause mortality or initiation of renal replacement therapy. **Results:** CRS-2 was identified in 130 patients (65.0%) and was associated with higher in-hospital mortality (32.3% vs. 11.4%, *p* = 0.002) and three-month mortality (44.6% vs. 21.4%, *p* = 0.002). Within the CRS-2 subgroup, patients who experienced the primary composite outcome had higher admission levels of cystatin C and urinary albumin-to-creatinine ratio (UACR) and lower estimated glomerular filtration rate (eGFR). ROC analysis demonstrated moderate discriminative ability of cystatin C (AUC 0.739) and UACR (AUC 0.733). In Cox regression analysis, cystatin C (HR 1.534, 95% CI 1.263–1.863, *p* < 0.001) and UACR (HR 1.003, 95% CI 1.001–1.006, *p* = 0.001) were significantly associated with the primary composite outcome. **Conclusions:** Renal dysfunction markers, particularly cystatin C and albuminuria, are associated with early adverse outcomes in CRS-2 patients hospitalized for decompensated HF. Routine assessment of these biomarkers may provide additional prognostic information and support risk assessment in this high-risk population.

## 1. Introduction

Chronic heart failure (HF) and chronic kidney disease (CKD) frequently coexist and interact through complex pathophysiological mechanisms rather than representing independent comorbid conditions [1]. Rather, persistent cardiac dysfunction may directly promote progressive renal injury through sustained haemodynamic impairment, maladaptive neurohormonal activation, inflammation, and oxidative stress [2]. This bidirectional interaction is conceptualized within the framework of cardiorenal syndromes (CRS), of which type 2 CRS (CRS-2) refers to chronic kidney dysfunction caused by HF [3]. The burden of both HF and CKD is substantial and continues to increase worldwide, driven by population ageing and the growing prevalence of hypertension, diabetes, obesity, and multimorbidity. In patients with chronic HF, CKD is highly prevalent, and renal dysfunction has repeatedly been shown to portend a greater therapeutic complexity, recurrent decompensation, and excess mortality [4]. However, renal involvement in HF is biologically heterogeneous and cannot be fully characterized by serum creatinine or estimated glomerular filtration rate (eGFR) alone. In the setting of CRS-2, kidney injury may reflect not only impaired filtration but also glomerular barrier damage, tubular-interstitial injury, endothelial dysfunction, congestion-related renal stress, and chronic inflammatory activation [5].

This complexity has generated increasing interest in renal biomarkers that capture complementary dimensions of kidney dysfunction [6]. Albuminuria reflects early glomerular injury and systemic endothelial dysfunction and may precede overt decline in eGFR. Cystatin C has emerged as a more sensitive marker of renal function than creatinine in many clinical settings, particularly where muscle mass, age, and sex may confound creatinine-based estimates [7]. Uric acid has also been linked to adverse outcomes in heart failure, likely reflecting impaired renal excretion, oxidative stress, and heightened xanthine oxidase activity [8]. Taken together, these markers may provide a more nuanced assessment of renal vulnerability in patients with decompensated chronic HF than conventional renal indices alone [9].

Despite the high prevalence of renal dysfunction in HF, CRS-2 remains relatively underexplored, particularly regarding the prognostic role of renal biomarkers in patients hospitalized with decompensated disease [10]. Therefore, the aim of this study was to evaluate the prognostic significance of renal biomarkers in patients with decompensated HF and CRS-2, with particular focus on their association with short-term adverse outcomes.

## 2. Materials and Methods

### 2.1. Study Design and Population

This prospective observational cohort study was conducted in the Intensive Care Unit of the Clinic for Internal Medicine at the University Clinical Center Tuzla, Bosnia and Herzegovina. Consecutive adult patients hospitalized for decompensated chronic heart failure were prospectively enrolled between 30 April 2025 and 31 October 2025. Included patients were adults (age ≥ 18 years) previously diagnosed with HF confirmed by clinical evaluation and echocardiographic findings, and index hospitalization was due to signs and symptoms of HF decompensation. Exclusion criteria included end-stage renal disease requiring chronic dialysis, severe chronic liver disease, active malignancy or autoimmune disease requiring immunosuppressive therapy, HF with preserved ejection fraction (HFpEF), and acute kidney disease.

#### 2.1.1. Definition of Cardiorenal Syndrome Type 2

CRS-2 was defined as the coexistence of HF and CKD, with renal dysfunction developing in the setting of long-standing heart failure, in accordance with the Kidney Disease: Improving Global Outcomes (KDIGO) criteria [11]. CKD was defined as reduced eGFR (<60 mL/min/1.73 m^2^) or the presence of at least one marker of kidney damage persisting for at least three months. HF was defined according to the 2022 AHA/ACC/HFSA heart failure guidelines based on left ventricular ejection fraction (LVEF) [12]. Only patients with HFrEF and HFmrEF were included in the final analysis. Based on longitudinal clinical documentation, including prior laboratory measurements and medical records, rather than solely on admission findings, patients were stratified into two groups. The CRS-2 group consisted of patients with HF and established CKD in whom HF preceded the development of renal dysfunction. The temporal relationship between heart failure and renal dysfunction was established through a detailed review of prior medical records, including previous laboratory measurements and clinical documentation. Only patients with documented evidence of chronic heart failure preceding the onset of chronic kidney disease were classified as having CRS-2. Patients with unclear temporal relationships or potential alternative causes of renal dysfunction were classified as non-CRS-2. The non-CRS-2 group included patients with HF who did not meet these criteria, including patients without CKD, as well as those in whom renal dysfunction could not be attributed to HF. Importantly, classification was not based solely on admission findings, but on longitudinal clinical documentation, including prior laboratory measurements and medical records.

#### 2.1.2. Clinical Management

All patients received guideline-directed medical therapy for HF in accordance with contemporary international recommendations.

### 2.2. Laboratory Measurements

Venous blood samples were obtained at hospital admission using K2E (ethylenediaminetetraacetic acid) anticoagulant tubes. Complete blood count parameters were analyzed using the Sysmex XN-1000 automated hematology analyzer (Sysmex Corporation, Kobe, Japan) according to the manufacturer’s calibration protocol.

Biochemical analyses were performed using the Beckman Coulter DxC 700 AU biochemical analyzer (Beckman Coulter Diagnostics, Brea, CA, USA). Serum creatinine, urea, cystatin C, and albumin concentrations were measured using standardized laboratory methods. Estimated glomerular filtration rate (eGFR) was calculated using the Chronic Kidney Disease Epidemiology Collaboration (CKD-EPI) equation. Urinary albumin excretion was assessed using the urine albumin-to-creatinine ratio (UACR) obtained from a spot urine sample collected at hospital admission. All laboratory measurements were performed in the central hospital laboratory according to standardized operating procedures as part of routine clinical care.

### 2.3. Echocardiographic Assessment

Transthoracic echocardiography was performed using a Vivid T8 ultrasound system (General Electric Medical Systems, Chicago, IL, USA; manufactured in Jiangsu, China) by experienced cardiologists who were blinded to the laboratory findings. LVEF was quantified using the biplane Simpson method in accordance with current echocardiographic guideline recommendations.

### 2.4. Clinical Follow-Up and Outcomes

All included patients were prospectively followed for 90 days after hospital admission according to the predefined study protocol. The primary outcome was defined as the occurrence of a composite endpoint consisting of all-cause mortality or initiation of renal replacement therapy during the follow-up period. Time-to-event was calculated from the date of hospital admission to the earliest of the first occurrence of the primary outcome or the end of the 90-day follow-up period.

### 2.5. Ethical Considerations

The study was conducted in accordance with the Declaration of Helsinki and approved by the Ethics Committee of the University Clinical Center Tuzla (protocol code 02-09/2-98-3/25, approved on 22 April 2025). Due to the observational design of the study and the use of routinely collected clinical data, the Ethics Committee waived the requirement for written informed consent.

### 2.6. Statistical Analysis

Statistical analysis was performed using IBM SPSS Statistics software (International Business Machines Corporation, Armonk, NY, USA; version 31) and the R software through the RStudio environment (Posit PBC, Boston, MA, USA; version 2025.09.0). Continuous variables were assessed for normality using the Shapiro-Wilk test with additional graphical inspection of distribution plots. As most variables demonstrated non-normal distributions, continuous variables are presented as the median with interquartile range (IQR), whereas categorical variables are expressed as the number (N) and percentage (%). Comparisons between groups were performed using the Mann-Whitney U test for continuous variables and the chi-square test or Fisher’s exact test for categorical variables, as appropriate.

Receiver operating characteristic (ROC) curve analysis was performed to evaluate the discriminative ability of selected renal biomarkers for predicting the primary composite outcome. ROC analyses were performed specifically within the CRS-2 subgroup to evaluate the discriminative ability of renal biomarkers for predicting the primary composite outcome. The area under the curve (AUC) and its corresponding 95% confidence interval (CI) were calculated. Optimal cutoff values were determined using the Youden index. Time-to-event analyses were performed using Kaplan-Meier survival curves, and differences between groups were assessed using the log-rank test. Associations between clinical and laboratory parameters and the primary composite outcome were evaluated using Cox proportional hazards regression models. The proportional hazards assumption was assessed using Schoenfeld residuals. Given the limited number of outcome events and the high degree of collinearity among renal biomarkers, multivariable Cox regression models were not performed. Instead, univariate analyses were used to explore associations between individual biomarkers and the outcome. Results are presented as hazard ratios (HRs) with corresponding 95% confidence intervals (CIs). A two-sided *p*-value < 0.05 was considered statistically significant.

## 3. Results

### 3.1. Study Population

A total of 200 consecutive patients hospitalized with decompensated chronic heart failure were included in the analysis. Cardiorenal syndrome type 2 (CRS-2) was identified in 130 patients (65.0%), while 70 patients (35.0%) did not meet CRS-2 criteria. Age and sex distribution did not differ significantly between the groups. Smoking was more frequent among patients without CRS-2 (65.7% vs. 45.4%, *p* = 0.006). At admission, patients with CRS-2 had significantly lower systolic and diastolic blood pressure and lower 24-h urine output compared with those without CRS-2 (systolic blood pressure: median 115 vs. 140 mmHg, *p* < 0.001; diastolic blood pressure: median 70 vs. 80 mmHg, *p* = 0.006; 24-h urine output: median 1200 vs. 1725 mL, *p* = 0.001). Baseline characteristics of the study population are presented in Table 1.

### 3.2. Echocardiographic Characteristics

Echocardiographic parameters did not differ significantly between patients with and without CRS-2. Left ventricular ejection fraction assessed by the Simpson method was comparable between groups (median 40% in both groups, *p* = 0.653). Left atrial diameter and inferior vena cava diameter were also similar (*p* = 0.404 and *p* = 0.169, respectively). The distribution of heart failure phenotypes (HFrEF, HFmrEF) did not differ between groups (*p* = 0.302). Detailed echocardiographic characteristics are presented in Table 2.

### 3.3. Renal Biomarkers and Urinalysis

Renal function markers were markedly impaired in patients with CRS-2 compared with those without CRS-2. Patients in the CRS-2 group had higher levels of urea (median 16.0 vs. 9.15 mmol/L, *p* < 0.001), creatinine (median 165.5 vs. 127.0 µmol/L, *p* < 0.001), cystatin C (median 2.49 vs. 1.55 mg/L, *p* < 0.001), and uric acid (median 571 vs. 457 µmol/L, *p* < 0.001). Estimated glomerular filtration rate (eGFR) was significantly lower in this group (median 31.7 vs. 44.6 mL/min/1.73 m^2^, *p* < 0.001).

Markers of albuminuria were also significantly higher in patients with CRS-2. The albumin-to-creatinine ratio (UACR) was higher in the CRS-2 group (median 20.82 vs. 7.19 mg/mmol, *p* < 0.001), as well as urinary albumin levels (median 136.5 vs. 69.5 mg/L, *p* < 0.001). Serum albumin levels were lower in patients with CRS-2 (median 39 vs. 41 g/L, *p* = 0.016). Detailed results of renal biomarkers and urinalysis are presented in Table 3.

### 3.4. Prognostic Value of Renal Biomarkers for Short-Term Outcomes

During the 90-day follow-up period, clinical outcomes differed substantially between patients with and without CRS-2. Renal replacement therapy was initiated more frequently in patients with CRS-2 than in those without CRS-2 (22.3% vs. 2.9%, *p* < 0.001). Ninety-day all-cause mortality was also higher among CRS-2 patients (44.6% vs. 21.4%, *p* = 0.002), and in-hospital mortality occurred more frequently in this group (32.3% vs. 11.4%, *p* = 0.002). The median time to the primary composite outcome, defined as all-cause mortality or initiation of renal replacement therapy, was significantly shorter in CRS-2 patients compared with those without CRS-2 (14.0 vs. 37.5 days, *p* = 0.020) (Table 4).

Within the CRS-2 subgroup, patients who experienced the primary composite outcome demonstrated significantly worse renal biomarker profiles at admission. Specifically, higher levels of urea, creatinine, cystatin C, and urinary albumin-to-creatinine ratio (UACR) were observed, while estimated glomerular filtration rate (eGFR) and serum albumin concentrations were significantly lower compared with patients without events (all *p* < 0.01) (Table 5).

**Figure 1:**
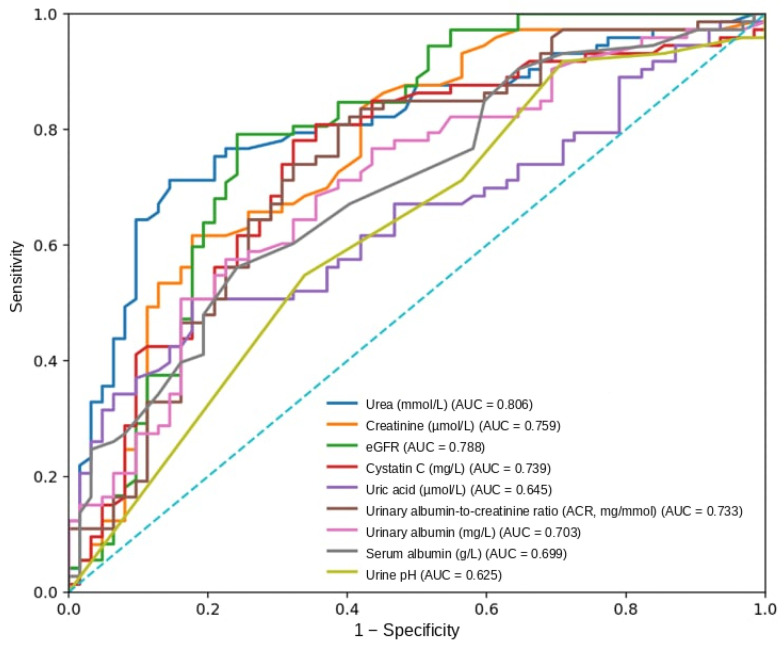
Receiver operating characteristic (ROC) curves demonstrating the predictive performance of selected renal biomarkers (urea, creatinine, eGFR, cystatin C, and UACR) for the three-month primary composite outcome in patients with cardiorenal syndrome type 2.

ROC analysis demonstrated moderate-to-good discriminative ability of renal biomarkers for predicting the primary composite outcome in patients with CRS-2. Urea showed the highest predictive performance (AUC 0.806), followed by eGFR (AUC 0.788), cystatin C (AUC 0.739), and UACR (AUC 0.733) (Table 6, Figure 1).

**Figure 2:**
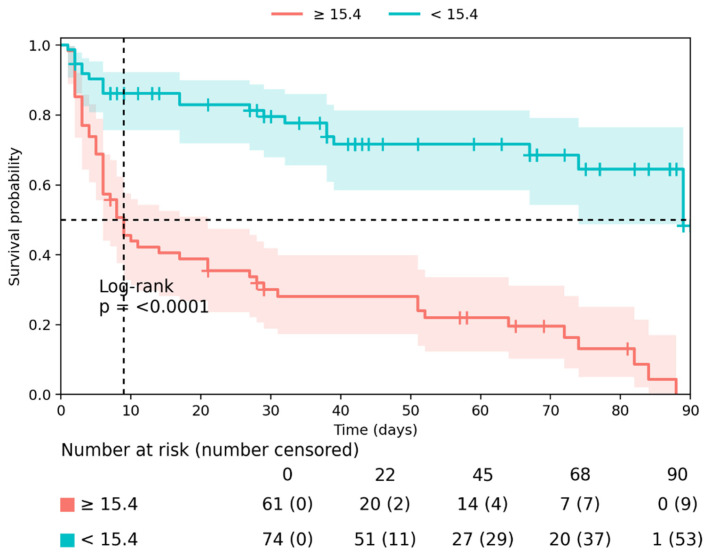
Kaplan-Meier survival curves for the primary composite outcome in patients with cardiorenal syndrome type 2, stratified by serum urea levels using a cut-off of 15.4 mmol/L, as determined by ROC analysis.

**Figure 3:**
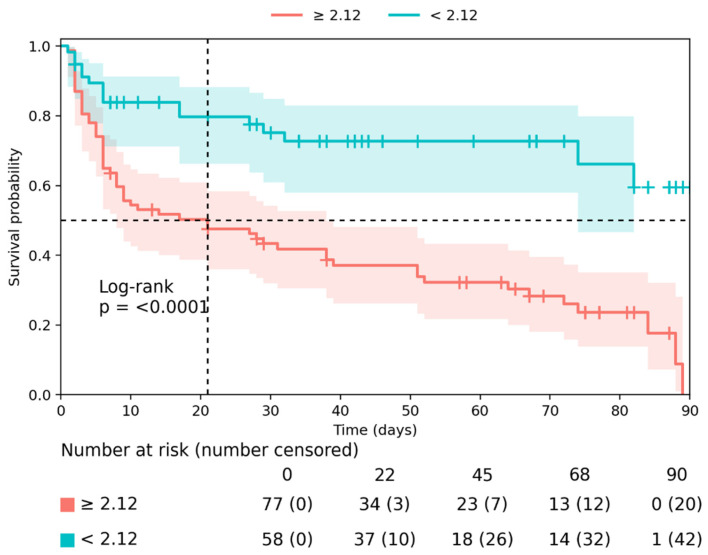
Kaplan-Meier survival curves for the primary composite outcome in patients with cardiorenal syndrome type 2, stratified by serum cystatin C levels using a cut-off of 2.12 mg/L, as determined by ROC analysis.

Kaplan-Meier survival analysis further confirmed the prognostic value of these biomarkers. Patients with abnormal renal biomarker values at admission had significantly higher cumulative incidence of the primary composite outcome during follow-up. In particular, cystatin C ≥ 2.12 mg/L, eGFR ≤ 37.2 mL/min/1.73 m^2^, UACR ≥ 10.49 mg/mmol, and urea ≥ 15.4 mmol/L were associated with significantly higher event rates (all log-rank *p* < 0.0001) (Figure 2 and Figure 3).

In Cox proportional hazards regression analysis, higher levels of urea (HR 1.044, 95% CI 1.029–1.059, *p* < 0.001), creatinine (HR 1.003, 95% CI 1.001–1.004, *p* = 0.002), cystatin C (HR 1.534, 95% CI 1.263–1.863, *p* < 0.001), and UACR (HR 1.003, 95% CI 1.001–1.006, *p* = 0.001) were associated with an increased risk of the primary composite outcome, whereas higher eGFR (HR 0.960, 95% CI 0.945–0.976, *p* < 0.001) and serum albumin concentrations (HR 0.932, 95% CI 0.898–0.967, *p* < 0.001) were associated with a reduced risk (Table 7). Hazard ratios are presented for a 1-unit increase in each variable (e.g., per 1 µmol/L increase for creatinine).

## 4. Discussion

This study demonstrates that CRS-2 is common among patients hospitalized with decompensated HF and is associated with markedly worse short-term outcomes [13]. Renal biomarkers measured at admission showed strong prognostic value, with cystatin C and albuminuria, reflected by the UACR, emerging as strong predictors of death or initiation of renal replacement therapy [14].

In this cohort, patients with CRS-2 exhibited significantly worse renal biomarker profiles than those without CRS-2, despite comparable echocardiographic characteristics and similar distributions of HF phenotypes. This observation is clinically relevant because it suggests that the excess risk observed in CRS-2 may not be fully explained by differences in cardiac structure or systolic function alone and is closely associated with concomitant renal dysfunction. In the context of HF, renal impairment reflects the cumulative effect of sustained haemodynamic compromise, renal venous congestion, and chronic neurohormonal activation involving the renin-angiotensin-aldosterone and sympathetic nervous systems, all of which contribute to progressive deterioration of kidney function. Similar observations have been reported in previous cohorts of patients with HF, where renal dysfunction has been shown to be strongly associated with outcomes, sometimes comparable to or exceeding the prognostic value of certain cardiac parameters in specific clinical contexts [15,16].

Renal biomarkers demonstrated substantial prognostic value for short-term outcomes. Patients with CRS-2 experienced significantly higher rates of mortality and renal replacement therapy initiation, and the median time to the composite outcome was considerably shorter compared with patients without CRS-2. Importantly, within the CRS-2 subgroup, individuals who developed adverse outcomes already had markedly worse renal biomarker profiles at admission. This finding suggests that renal dysfunction in CRS-2 is not merely a concurrent condition but is strongly associated with early clinical deterioration. This observation aligns with earlier studies showing that markers of renal impairment in heart failure are strongly associated with early mortality and rehospitalization, underscoring the prognostic importance of kidney function in the cardiorenal continuum [17,18].

Among the evaluated biomarkers, cystatin C and albuminuria showed the strongest associations with the primary composite outcome in Cox regression analysis. These findings highlight the complementary information provided by markers reflecting different aspects of kidney injury. Cystatin C primarily reflects reduced glomerular filtration and may detect subtle reductions in renal function earlier than creatinine-based estimates. In contrast, albuminuria reflects structural and functional damage to the glomerular filtration barrier and is often considered a marker of systemic endothelial dysfunction. These findings suggest that the combination of impaired filtration and glomerular injury identifies patients with particularly high vulnerability to adverse outcomes. Previous investigations have similarly demonstrated that cystatin C may provide superior prognostic information compared with creatinine-based estimates of renal function in patients with heart failure [19]. Likewise, albuminuria has been increasingly recognized as an independent predictor of cardiovascular and renal outcomes in HF populations, reflecting both renal injury and systemic vascular dysfunction [20].

ROC and Kaplan-Meier analyses further supported the prognostic relevance of renal biomarkers. Urea and eGFR showed the highest discriminative performance for predicting the composite outcome, while Kaplan-Meier analyses showed clear separation of event curves by biomarker thresholds. However, urea is a non-specific marker influenced by multiple factors, including catabolic state, volume status, and diuretic therapy. Therefore, its prognostic value should be interpreted cautiously and not considered a direct measure of intrinsic renal injury. These findings indicate that simple laboratory parameters obtained at hospital admission can provide clinically meaningful information regarding early risk in patients with CRS-2. Importantly, these results are consistent with prior studies suggesting that routinely available renal biomarkers can serve as practical tools for early risk stratification in hospitalized HF patients [21,22]. Taken together, the present results reinforce the concept that renal dysfunction represents a central component of the pathophysiological continuum linking HF and adverse outcomes. In CRS-2, kidney injury reflects the downstream consequences of chronic haemodynamic impairment and venous congestion, thereby integrating the cumulative burden of disease more directly than many other circulating biomarkers [23]. From a clinical perspective, early identification of patients with markedly abnormal renal biomarker profiles may allow improved risk stratification and potentially guide more intensive monitoring and therapeutic strategies during the vulnerable early phase following hospitalization. These findings suggest that renal biomarkers may complement, rather than replace, established clinical risk stratification approaches.

Several limitations should be acknowledged. Misclassification of CRS-2 cannot be fully excluded, as the temporal relationship between heart failure and renal dysfunction was established through a retrospective review of clinical records rather than a prospective longitudinal assessment. First, the single-center observational design may limit the generalizability of the findings. Second, although a consecutive cohort of hospitalized patients was included, the relatively limited number of outcome events may have affected the stability of regression estimates. Third, laboratory parameters were assessed only at hospital admission, and dynamic changes in renal biomarkers during hospitalization were not evaluated. Fourth, multivariable adjustment was not performed due to the limited number of outcome events and substantial collinearity among renal biomarkers, which limits the ability to establish independent prognostic value. Furthermore, internal validation was not performed, which may limit the generalizability of the predictive performance. The lack of established heart failure severity markers, such as NT-proBNP, NYHA functional class, congestion markers, and diuretic dose, limits the ability to assess whether renal biomarkers provide incremental prognostic value beyond standard cardiac risk markers and prevents assessment of their independence from heart failure severity. No formal adjustment for multiple comparisons was performed, which may increase the risk of type I error. Finally, the potential influence of diuretic therapy and fluid redistribution on laboratory parameters could not be fully controlled, and residual confounding related to unmeasured clinical variables cannot be excluded.

## 5. Conclusions

This study highlights the prognostic relevance of renal biomarkers in patients with cardiorenal syndrome type 2 hospitalized for decompensated chronic heart failure. Biomarkers reflecting impaired filtration and albuminuria, particularly cystatin C and the urinary albumin-to-creatinine ratio, were strongly associated with early adverse outcomes. The incremental prognostic value over established risk models was not assessed. These findings suggest that routine assessment of renal biomarkers may provide additional prognostic information and could support risk assessment in this high-risk population, although their incremental value beyond established clinical models remains to be determined.

## Figures and Tables

**Table 1:** Baseline clinical and demographic characteristics of the study population.

Variable	Total (N = 200)	CRS-2 (N = 130)	No CRS-2 (N = 70)	*p* Value
Sex, *n* (%)				0.142
Male	103 (51.5)	62 (47.7)	41 (58.6)	
Female	97 (48.5)	68 (52.3)	29 (41.4)	
Age, years, median (IQR)	71.0 (65.5–81.0)	72.0 (68.0–82.0)	69.5 (63.0–78.0)	0.098
Current smoker, *n* (%)	105 (52.5)	59 (45.4)	46 (65.7)	0.006
Hypertension, *n* (%)	193 (96.5)	124 (95.4)	69 (98.6)	0.242
Type 2 diabetes mellitus, *n* (%)	108 (54.0)	75 (57.7)	33 (47.1)	0.153
Hyperlipidemia, *n* (%)	197 (98.5)	127 (97.7)	70 (100.0)	0.200
Alcohol consumption, *n* (%)	58 (29.0)	38 (29.2)	20 (28.6)	0.922
Family history of cardiovascular disease, *n* (%)	195 (97.5)	125 (96.2)	70 (100.0)	0.097
Family history of chronic kidney disease, *n* (%)	97 (48.5)	66 (50.8)	31 (44.3)	0.382
Body weight (kg), median (IQR)	88.0 (85.0–92.0)	88.0 (85.0–92.0)	89.0 (85.0–93.0)	0.698
Height (cm), median (IQR)	169.0 (163.0–175.0)	168.0 (163.0–175.0)	170.5 (165.0–175.0)	0.209
Systolic blood pressure (mmHg), median (IQR)	130.0 (100.0–147.5)	115.0 (100.0–140.0)	140.0 (110.0–150.0)	<0.001
Diastolic blood pressure (mmHg), median (IQR)	70.0 (60.0–90.0)	70.0 (60.0–90.0)	80.0 (70.0–90.0)	0.006
Heart rate (beats/min), median (IQR)	110.0 (95.0–120.0)	110.0 (95.0–120.0)	110.0 (100.0–115.0)	0.322
24-h urine output (mL), median (IQR)	1400 (850–2100)	1200 (600–2000)	1725 (1200–2400)	0.001

Abbreviations: CRS-2, cardiorenal syndrome type 2; IQR, interquartile range; CKD, chronic kidney disease.

**Table 2:** Echocardiographic characteristics of the study population.

Variable	Total (N = 200)	CRS-2 (N = 130)	No CRS-2 (N = 70)	*p* Value
Left ventricular ejection fraction (%)—Simpson method, median (IQR)	40.0 (30.0–40.0)	40.0 (30.0–45.0)	40.0 (30.0–40.0)	0.653
Left atrial diameter (mm), median (IQR)	46.0 (43.0–50.0)	46.0 (43.0–50.0)	47.5 (43.0–52.0)	0.404
Inferior vena cava diameter (mm), median (IQR)	20.0 (18.0–23.0)	20.0 (19.0–23.75)	20.0 (18.0–23.0)	0.169

Abbreviations: CRS-2, cardiorenal syndrome type 2; LV, left ventricle; IQR, interquartile range; IVC, inferior vena cava.

**Table 3:** Renal biomarkers and urinalysis findings in the study population.

Variable	Total (N = 200)	CRS-2 (N = 130)	No CRS-2 (N = 70)	*p* Value
Renal function tests				
Urea (mmol/L), median (IQR)	12.30 (9.00–20.30)	16.00 (10.90–25.90)	9.15 (7.40–11.50)	<0.001
Creatinine (µmol/L), median (IQR)	139.50 (125.50–191.00)	165.50 (134.00–234.00)	127.00 (122.00–137.00)	<0.001
Estimated glomerular filtration rate (eGFR, mL/min/1.73 m^2^), median (IQR)	37.20 (26.30–48.80)	31.70 (19.50–41.30)	44.55 (39.30–53.60)	<0.001
Cystatin C (mg/L), median (IQR)	2.10 (1.49–3.00)	2.49 (1.84–3.19)	1.55 (1.28–1.98)	<0.001
Uric acid (µmol/L), median (IQR)	534.00 (429.50–634.00)	571.00 (445.00–672.00)	457.00 (385.00–582.00)	<0.001
Urinalysis parameters				
Urinary albumin-to-creatinine ratio (UACR, mg/mmol), median (IQR)	13.89 (6.28–49.01)	20.82 (9.52–84.85)	7.19 (3.68–16.42)	<0.001
Urinary albumin (mg/L), median (IQR)	91.00 (44.50–247.00)	136.50 (60.00–359.00)	69.50 (33.00–127.00)	<0.001
Urinary creatinine (mmol/L), median (IQR)	7.78 (3.48–12.57)	6.28 (3.57–11.34)	9.50 (3.21–17.25)	0.100
Serum albumin (g/L), median (IQR)	40.00 (35.00–44.00)	39.00 (34.00–42.00)	41.00 (36.00–45.00)	0.016
Urine pH, median (IQR)	5.50 (5.00–6.00)	5.00 (5.00–6.00)	5.50 (5.00–6.00)	0.029
Urine specific gravity (kg/L), median (IQR)	1.02 (1.01–1.02)	1.02 (1.01–1.02)	1.02 (1.01–1.02)	0.641
Urobilinogen (µmol/L), median (IQR)	16.00 (16.00–16.00)	16.00 (16.00–16.00)	16.00 (16.00–16.00)	0.897
Haptoglobin (g/L), median (IQR)	2.44 (1.12–3.11)	2.55 (1.11–3.14)	2.34 (1.14–2.99)	0.284

Abbreviations: CRS-2, cardiorenal syndrome type 2; IQR, interquartile range; eGFR, estimated glomerular filtration rate; UACR, albumin-to-creatinine ratio.

**Table 4:** Short-term clinical outcomes according to the presence of cardiorenal syndrome type 2.

Outcome Variable	Total (N = 200)	CRS-2 (N = 130)	No CRS-2 (N = 70)	*p* Value
Length of hospital stay (days), median (IQR)	10.00 (7.00–12.00)	9.50 (7.00–12.00)	10.00 (8.00–12.00)	0.310
Hemodialysis during hospitalization, *n* (%)	31 (15.5)	29 (22.3)	2 (2.9)	<0.001
3-month mortality, *n* (%)	73 (36.5)	58 (44.6)	15 (21.4)	0.002
In-hospital mortality, *n* (%)	50 (25.0)	42 (32.3)	8 (11.4)	0.002
Time to primary composite outcome (days), median (IQR)	27.00 (6.00–58.50)	14.00 (6.00–51.00)	37.50 (13.25–68.00)	0.020

Abbreviations: CRS-2, cardiorenal syndrome type 2; IQR, interquartile range.

**Table 5:** Renal biomarkers and urinalysis parameters according to the occurrence of the primary composite outcome in patients with CRS-2.

Parameter	Primary Composite Outcome	No Primary Composite Outcome	*p* Value
Renal function parameters			
Urea (mmol/L), median (IQR)	20.30 (13.90–28.80)	10.35 (7.80–12.70)	<0.001
Creatinine (µmol/L), median (IQR)	180 (138–237)	131 (123–155.75)	<0.001
Estimated glomerular filtration rate (eGFR, mL/min/1.73 m^2^), median (IQR)	30.10 (19.38–36.30)	46.10 (38.40–54.98)	<0.001
Serum albumin (g/L), median (IQR)	38 (31–41)	41 (39–45)	<0.001
Cystatin C (mg/L), median (IQR)	2.85 (2.12–3.55)	1.69 (1.31–2.38)	<0.001
Uric acid (µmol/L), median (IQR)	622 (439–776)	506 (428.75–605)	0.004
Urinalysis parameters			
Urinary albumin-to-creatinine ratio (UACR, mg/mmol), median (IQR)	28.34 (12.79–101.25)	7.99 (2.92–20.37)	<0.001
Urinary albumin (mg/L), median (IQR)	163 (73–365)	68 (31–132.75)	<0.001
Urinary creatinine (mmol/L), median (IQR)	5.93 (3.25–10.11)	8.61 (3.36–16.66)	0.085
Urine pH, median (IQR)	5 (5–6)	5.50 (5–6.50)	0.008
Urine specific gravity (kg/L), median (IQR)	1.02 (1.01–1.02)	1.02 (1.01–1.02)	0.185
Urobilinogen (µmol/L), median (IQR)	16 (16–16)	16 (16–16)	0.121
Haptoglobin (g/L), median (IQR)	2.74 (0.55–3.41)	2.23 (1.15–2.99)	0.213

Abbreviations: CRS-2, cardiorenal syndrome type 2; eGFR, estimated glomerular filtration rate; UACR, albumin-to-creatinine ratio; IQR, interquartile range.

**Table 6:** Receiver operating characteristic (ROC) analysis of renal biomarkers for predicting the three-month primary composite outcome.

Parameter	Cut-Off Value	AUC (95% CI)	*p* Value
Renal function tests			
Urea (mmol/L)	≥15.40	0.806 (0.614–0.999)	0.002
Creatinine (µmol/L)	≥164.00	0.759 (0.555–0.964)	0.013
Estimated glomerular filtration rate (eGFR, mL/min/1.73 m^2^)	≤37.20	0.788 (0.589–0.988)	0.005
Cystatin C (mg/L)	≥2.12	0.739 (0.538–0.940)	0.020
Serum albumin (g/L)	≤38.00	0.699 (0.495–0.902)	0.055
Uric acid (µmol/L)	≥622.00	0.645 (0.432–0.857)	0.182
Urinalysis parameters			
Urinary creatinine (mmol/L)	≤13.65	0.586 (0.365–0.808)	0.444
Urinary albumin-to-creatinine ratio (UACR, mg/mmol)	≥10.49	0.733 (0.525–0.941)	0.028
Urinary albumin (mg/L)	≥136.00	0.703 (0.497–0.909)	0.053
Urine pH	≤5.00	0.625 (0.415–0.835)	0.242

Abbreviations: AUC, area under the curve; CI, confidence interval; eGFR, estimated glomerular filtration rate; UACR, albumin-to-creatinine ratio.

**Table 7:** Univariate Cox proportional hazards regression analysis of renal biomarkers and urinalysis parameters for prediction of the primary composite outcome in patients with cardiorenal syndrome type 2.

Parameter	HR	95% CI	*p* Value
Renal function parameters			
Urea (mmol/L)	1.044	1.029–1.059	<0.001
Creatinine (µmol/L)	1.003	1.001–1.004	0.002
Estimated glomerular filtration rate (eGFR, mL/min/1.73 m^2^)	0.960	0.945–0.976	<0.001
Cystatin C (mg/L)	1.534	1.263–1.863	<0.001
Serum albumin (g/L)	0.932	0.898–0.967	<0.001
Uric acid (µmol/L)	1.002	1.001–1.004	<0.001
Urinalysis parameters			
Urinary creatinine (mmol/L)	2.726	1.416–5.247	0.002
Urinary albumin-to-creatinine ratio (UACR, mg/mmol)	1.003	1.001–1.006	0.001
Urinary albumin (mg/L)	1.001	1.000–1.001	0.001
Urine pH	0.770	0.560–1.060	0.109

Abbreviations: HR, hazard ratio; CI, confidence interval; eGFR, estimated glomerular filtration rate; UACR, albumin-to-creatinine ratio.

## Data Availability

The original contributions presented in this study are included in the article. Further inquiries can be directed to the corresponding author.

## Abbreviations

ACR	Albumin-to-creatinine ratio
AUC	Area under the curve
CI	Confidence interval
CKD	Chronic kidney disease
CRS-2	Cardiorenal syndrome type 2
eGFR	Estimated glomerular filtration rate
HF	Heart failure
HFmrEF	Heart failure with mildly reduced ejection fraction
HFrEF	Heart failure with reduced ejection fraction
HFpEF	Heart failure with preserved ejection fraction
HR	Hazard ratio
IQR	Interquartile range
KDIGO	Kidney Disease: Improving Global Outcomes
ROC	Receiver operating characteristic
UACR	Urinary albumin-to-creatinine ratio
